# Recurrent spinal atypical teratoid/rhabdoid tumor with pulmonary metastasis

**DOI:** 10.1002/cnr2.1975

**Published:** 2024-01-13

**Authors:** Akinori Yaguchi, Junya Fujimura, Kazutaka Maruyama, Megumi Fujiwara, Takeshi Ishibashi, Osamu Tomita, Toshiaki Shimizu

**Affiliations:** ^1^ Department of Pediatrics Juntendo University Faculty of Medicine Tokyo Japan

**Keywords:** atypical teratoid/rhabdoid tumor, metastatic recurrence, multidisciplinary management, pulmonary metastasis, spinal cord

## Abstract

**Background:**

Atypical teratoid/rhabdoid tumors (ATRT) are aggressive pediatric central nervous system malignancies that predominantly affect the brain and have poor survival outcomes. However, spinal ATRT is an uncommon subset of ATRT, and its clinical course and management are poorly understood.

**Case:**

We describe a case of spinal ATRT in a previously healthy 5‐year‐old girl who initially presented with rapid‐onset gait disturbance. Magnetic resonance imaging (MRI) revealed an extramedullary tumor at thoracic level 5 (T5) without bony destruction or metastasis. The patient partially recovered after surgical resection. One month was required for a definitive diagnosis, and the pathology confirmed ATRT characterized by the loss of INI‐1 protein expression. Chemoradiotherapy with local irradiation and high‐dose chemotherapy with autologous peripheral blood stem cell transplantation led to complete remission and functional recovery for 5 months. However, the condition exhibited progression in the cerebrospinal fluid (CSF) region, resulting in cerebellar, cerebral, and spinal tumor development. Eventually, the disease metastasized to the lungs and disseminated to the entire cerebrospinal cord and fluid. The patient died 15 months after the initial diagnosis.

**Conclusion:**

This case emphasizes the importance of considering ATRT as a potential diagnostic modality for pediatric spinal cord tumors, enabling prompt multidisciplinary intervention. The heterogeneous appearance of spinal ATRT may make distinguishing it from other spinal tumors difficult, resulting in delayed diagnosis and treatment. The treatment approach for ATRT remains challenging with no established standards. Local irradiation may be preferable to minimize neurodevelopmental effects, and initial craniospinal irradiation may potentially prevent recurrence. Our case emphasizes the likelihood of extracranial metastasis in ATRT, thereby highlighting the importance of a comprehensive assessment of both genetic and epigenetic profiles to identify any factors that may influence the clinical course of this disease. Prompt diagnosis and comprehensive therapeutic strategies are critical for improving outcomes in spinal ATRT patients.

## 1 INTRODUCTION

Atypical teratoid/rhabdoid tumors (ATRTs) are aggressive malignant tumors of the pediatric central nervous system (CNS) with poor survival outcomes.^1^, ^2^, ^3^ Common sites of primary pediatric ATRTs are the supratentorial and infratentorial compartments; spinal ATRTs occur in 1%–2% of all ATRT cases, and there are limited data regarding primary spinal ATRTs and extra‐CNS metastasis.^2^, ^4^, ^5^, ^6^ A report from the Children's Oncology Group (COG) trial ACN033 included one spinal cord ATRT in 65 patients,^2^ and the results from the St. Jude multi‐institutional trial SJMB03 included 2 spinal cord ATRTs in 22 patients.^4^ In Japan, a retrospective study by the Japan Children's Cancer Group included 1 spinal cord ATRT in 38 patients.^1^ The radiographic appearance of spinal ATRT is heterogeneous and can be confused with more common spinal tumors, such as nerve sheath tumors.^7^ Histological features of ATRT can also be heterogeneous. ATRT is typically characterized by loss of expression of *SMARCB1* gene product integrase interactor 1 (INI‐1) protein.^8^, ^9^ Herein, we present a rare case of spinal ATRT in which an extramedullary tumor was suspected. The diagnosis was based on the loss of INI‐1 levels, and multidisciplinary therapy with focal RT was temporarily effective. However, the patient repeatedly displayed recurrence at non‐irradiated sites and developed pulmonary metastases. Consideration of ATRT for pediatric spinal cord tumors is important because of the need for prompt multidisciplinary treatment. In addition, spinal cord ATRT may metastasize outside the central nervous system, such as the lungs; therefore, a systemic search may be important at the first onset, recurrence, and follow‐up.

## 2 CASE

**FIGURE 1:**
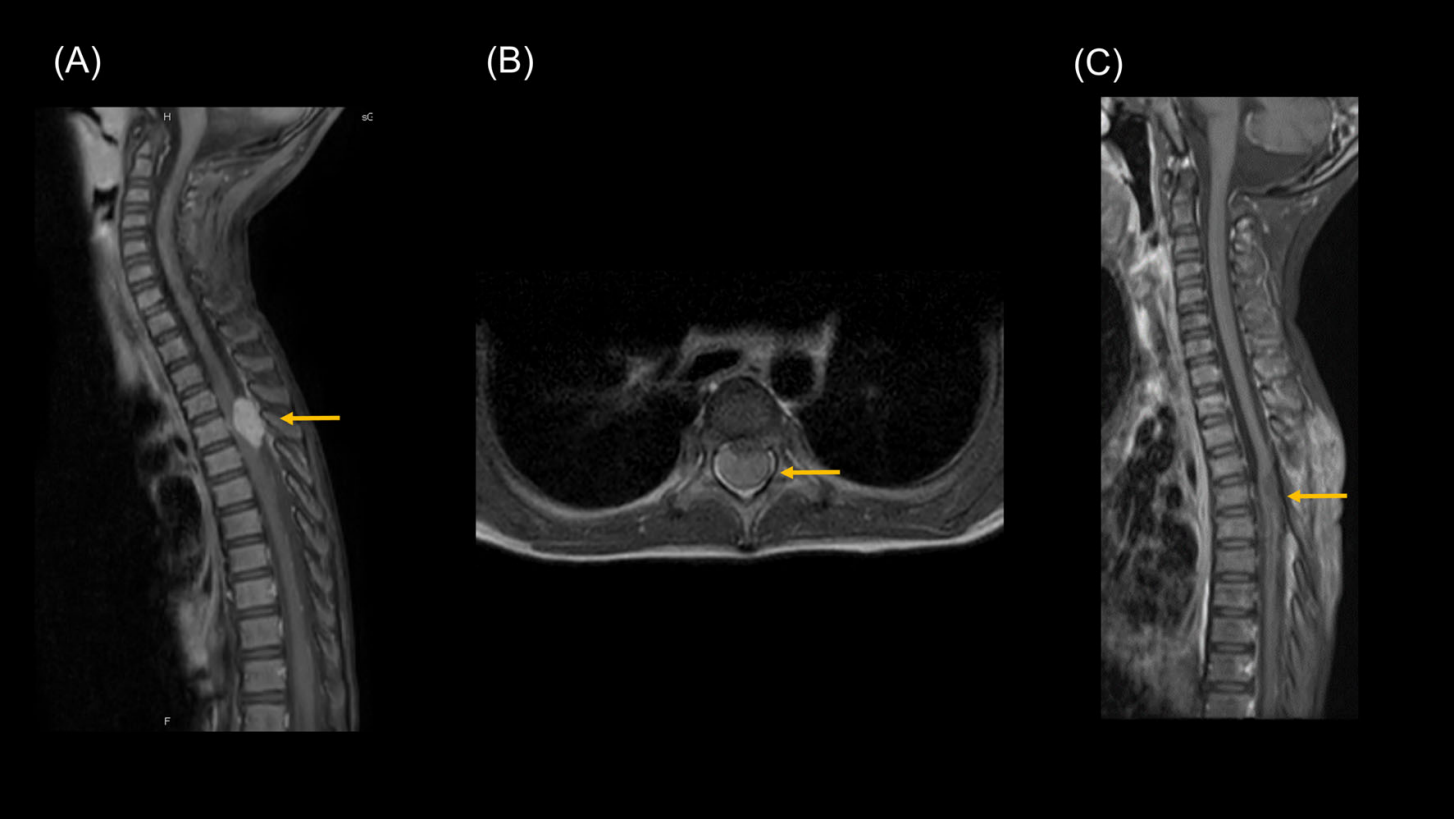
Gadolinium‐enhanced T1 weighted (Gd‐T1w) magnetic resonance imaging (MRI) at diagnosis. (A) The sagittal section revealed a mass with contrast enhancement at the T5 level. (B) The mass appeared relatively distinct from the spinal cord in the axial section. (C) Post‐removal, post‐radiochemotherapy, and pre‐high‐dose chemotherapy images show a good response to treatment.

A previously healthy 5‐year‐old Japanese girl presented with gait disturbance that rapidly progressed over 4 days. She could not stand and was referred to the Juntendo University Hospital in August 2021. The results of the physical examination were as follows: manual muscle testing of the hip joint was 2/3, knee extension was 2/3, ankle dorsiflexion was 2/4, deep tendon reflex of the kneecap was +/+, Achilles tendon was −/−, Babinski reflex was +/+, and superficial limb sensory examination revealed +/+ results for both the right and left sides. Additionally, the right and left sides demonstrated severe dullness, and bladder–rectal disturbances were present. Magnetic resonance imaging (MRI) of the spine revealed an extramedullary tumor at T5 without bony destruction or metastasis (Figure 1A,B). Preoperative differential diagnoses by a radiologist included ependymoma or schwannoma. The tumor was almost completely removed macroscopically by the surgeon; however, adhesions to the nerves were prominent. Intraoperative findings indicated that the tumor was extramedullary. The patient regained the ability to walk with assistance. We awaited pathology results to determine additional treatments. One month after surgery, the patient experienced symptoms of recurrence, including lower‐extremity sensory deficits and bladder–rectal disturbances. We performed cranial and spinal MRI, which confirmed an enlarged tumor in the same region without metastases. Pathological analysis revealed ATRT at the time. The tumor comprised small cells with indistinct differentiation, pale nuclei with distinct nucleoli, and vacuoles. The cells expressed various cell lineage markers such as vimentin and synaptophysin, and INI‐1 expression was lost in tumor cell nuclei. Chemoradiotherapy was promptly initiated according to the European Rhabdoid Registry (EU‐RHAB) protocol.^6^, ^10^ Radiotherapy was started immediately; therefore, we followed the protocol and administered a single methotrexate intrathecal injection before the start of radiotherapy. Cerebrospinal fluid cytology revealed no malignant findings; hence, we decided to administer local extended tumor region irradiation by photon stereotactic radiotherapy (SRT) with 54.0 Gy (1.8 Gy/day, five fractions/week). One month later, MRI revealed that the tumor had disappeared (Figure 1C) and complete remission (CR) was achieved. Consequently, we administered high‐dose chemotherapy with carboplatinum (500 mg/m^2^ from day −6 to day −4) and thiotepa (300 mg/m^2^ from day −6 to day −4) followed by autologous peripheral blood stem cell transplantation (day 0) based on EU‐RHAB.^6^, ^10^ The patient completed the treatment without any adverse events other than hematological toxicity and remained in CR. The patient regained the ability to walk unassisted through rehabilitation intervention, and her bladder–rectal disturbances were resolved.

**FIGURE 2:**
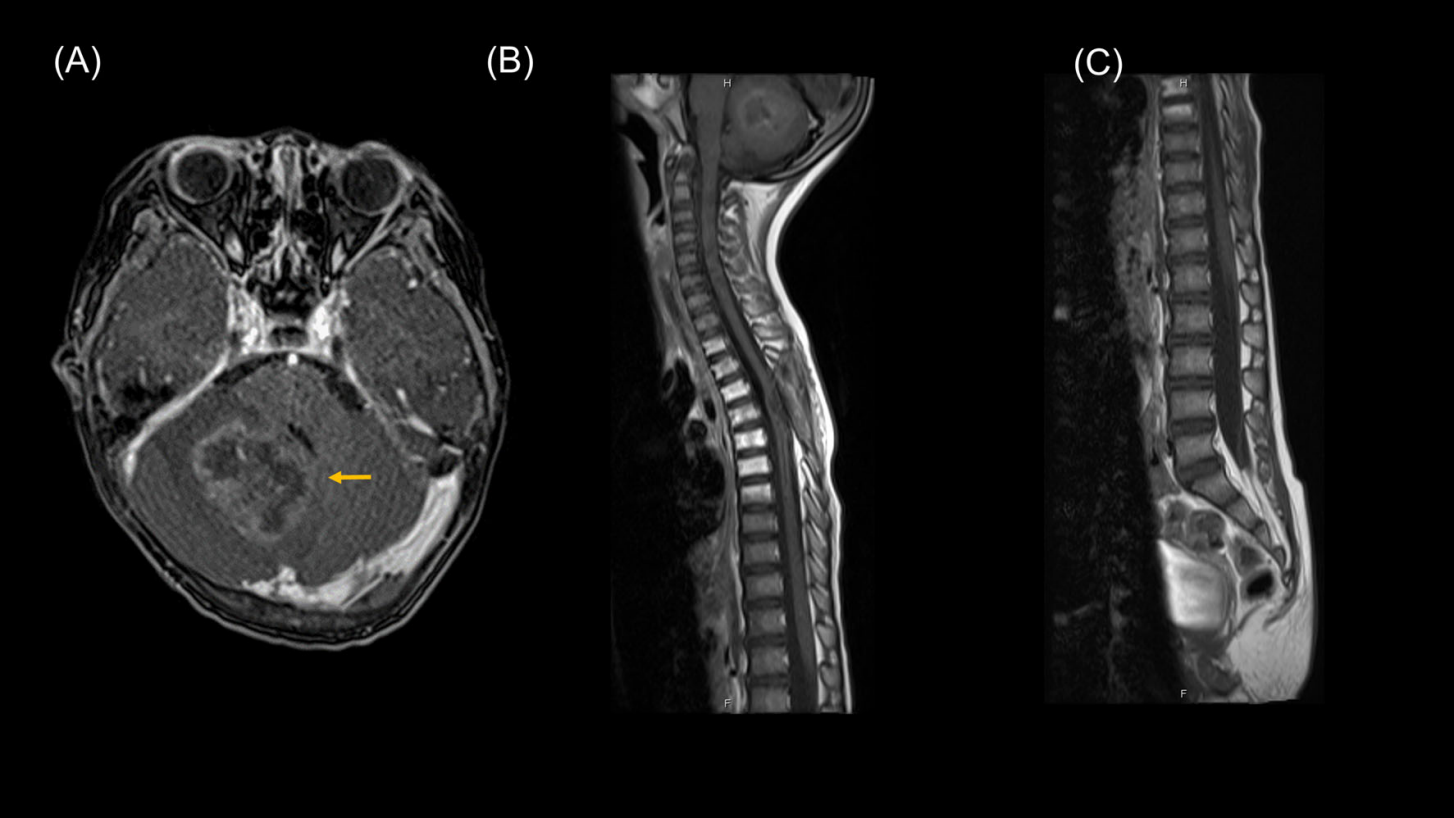
Gd‐T1w MRI at first relapse in the cerebellum. No new lesions were observed in the spinal cord. Subsequently, gross total resection was performed, and local radiotherapy was administered.

The patient remained in CR and attended school unassisted for 5 months. However, the patient developed an ataxic gait, and MRI revealed cerebellar recurrence (Figure 2A). No recurrence was observed in the spinal cord (Figure 2B,C). The neurosurgeons completely resected the tumor macroscopically. Postoperative MRI resonance imaging confirmed that the tumor had been completely removed. Regarding additional treatment, chemotherapy was considered ineffective because of early recurrence after chemotherapy, including high‐dose chemotherapy. Regarding radiotherapy, the radiologist determined that re‐irradiation of the already irradiated area was associated with a high risk of neurological necrosis; therefore, local irradiation of 54.0 Gy (1.8 Gy/day, five fractions/week) by intensity‐modulated radiation therapy (IMRT) at the tumor bed of the cerebellum was performed.

**FIGURE 3:**
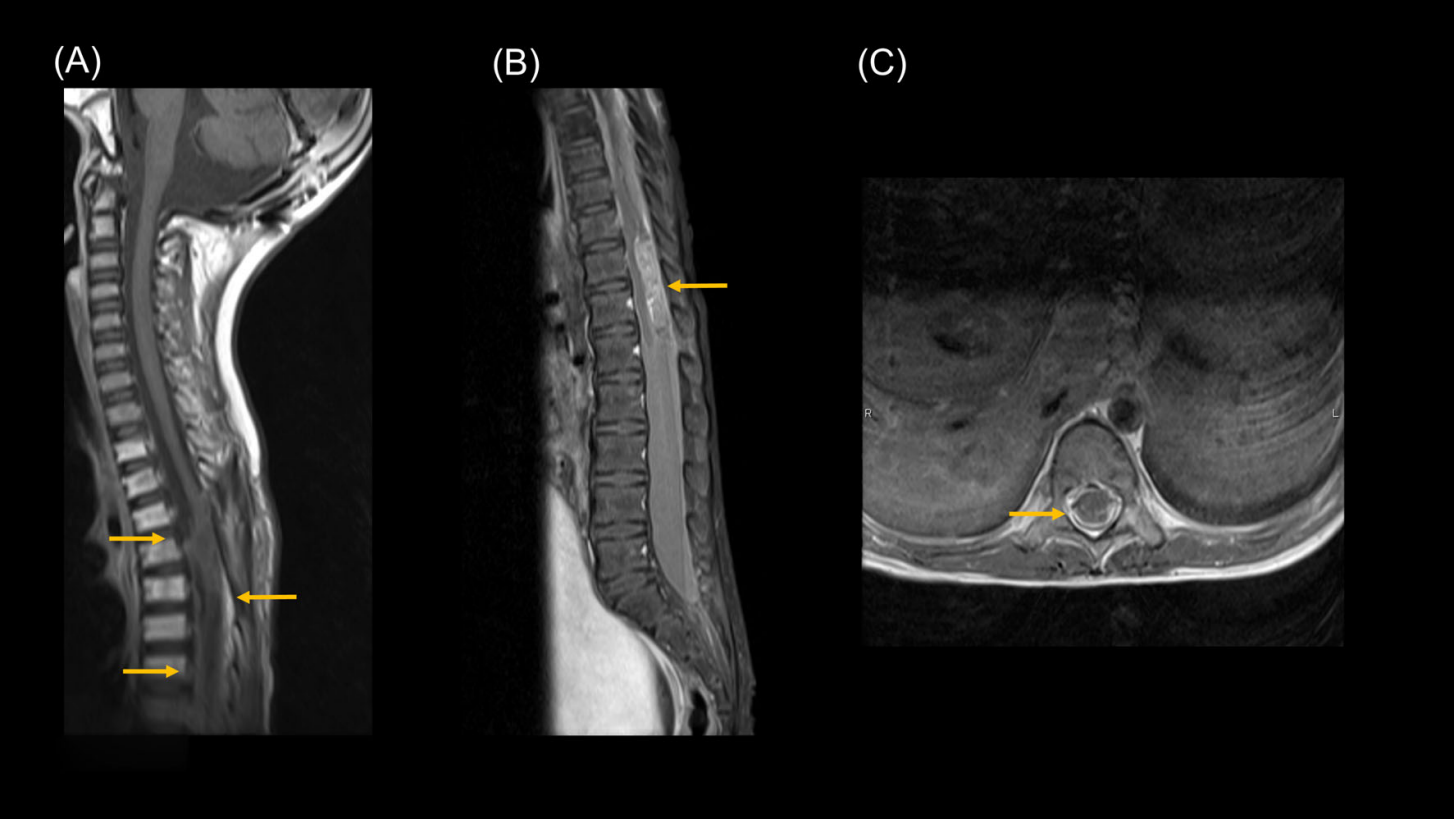
Contrast effects on the spinal cord limbus caudal to the T6 vertebral level (A) (B); a mass was present at the T10 to L1 level (B). The caudal nerve was enlarged and had a limbic enhancing effect (C), suggesting a disseminated lesion caudal to the level of the T6 vertebral body.

The patient regained the ability to walk unassisted; however, 1 month after RT, she developed pain and paresthesia in the lower extremities. Magnetic resonance imaging (MRI) revealed recurrence at a non‐irradiated site in the lumbar spinal cord (mass lesion at T10‐L1 and contrast effect at T6 to cauda equina; Figure 3). Local irradiation with SRT photons (20.0 Gy) was administered at the T12–L3 level for symptom relief. The patient's lower extremity pain and sensory disturbances resolved, but her ataxic gait persisted.

**FIGURE 4:**
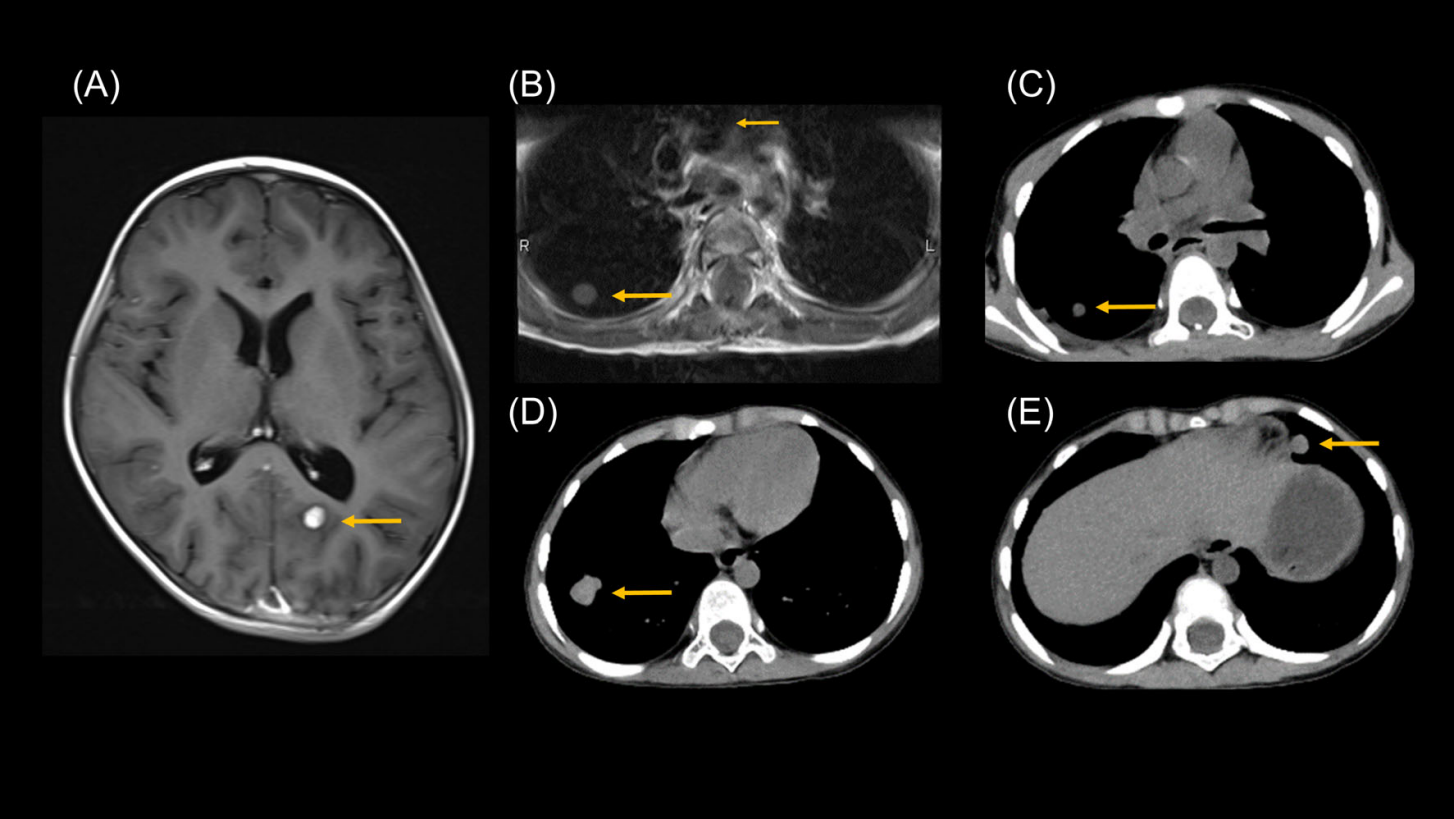
After irradiating the mass in Figure 3, Gd‐T1w MRI showed a 6 mm contrast‐enhanced nodule in the left parieto‐occipital lobe (A). Spinal MRI incidentally revealing a lung lesion (B). Whole‐body computed tomography (CT) was performed to search for metastases, and multiple masses were found in the lungs (C)–(E). There were no lesions in other body parts, including the kidneys and liver.

One month later, follow‐up MRI revealed recurrence in the cerebrum (Figure 4A), and spinal cord MRI incidentally revealed lung metastases (Figure 4B). There were no lesions in the lungs as far as they could be observed on MRI at the time of the initial onset and at the time of the first recurrence, and there were no lesions in the kidneys or liver. The patient had no family history of malignancy among her grandparents, parents, or brothers. Computed tomography revealed multiple lung tumors but no other affected sites (Figure 4C–E). We considered this to be pulmonary metastasis of ATRT. At the parent's request, we performed tumor resection of the right lower lung to differentiate between a benign tumor, a second cancer, and ATRT metastasis. The tumor was composed of undifferentiated small cells with a high nuclear‐to‐cytoplasmic ratio similar to the initial presentation. Extensive necrosis was observed in the tumor. INI‐1 was negative. The patient could play and perform personal care in a sitting position but could not stand alone. We considered the possibility of enrolling the patient in a phase I study of enhancer of zeste homolog (EZH) 1/2 dual inhibitors, which are considered effective for malignant rhabdoid tumors,^11^ but decided against it because the patient did not meet the eligibility criteria. The patient's Lansky score was less than 50 because the patient could not stand by herself.

**FIGURE 5:**
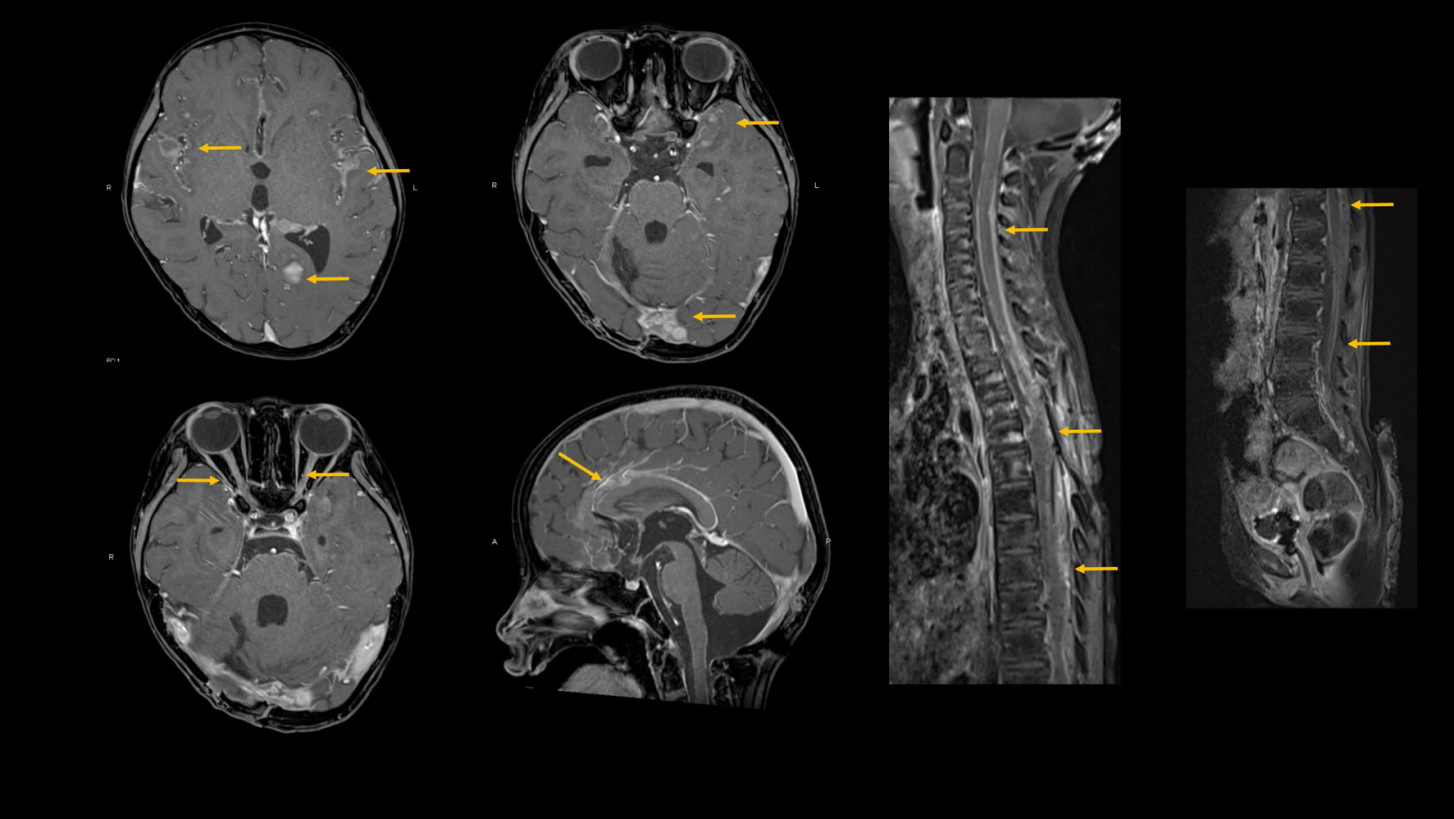
Gd‐T1w MRI revealed whole cerebrospinal dissemination and multiple metastases. Head MRI showed extensive contrast enhancement of the leptomeningeal membrane, with enhancement effects along the bilateral optic nerves. Spinal MRI revealed a diffuse enhancement effect within the spinal canal. There was an enhancing mass in the postoperative area of the upper thoracic spine and a continuous enhancing effect along the surface of the spinal cord to the spinal conchal level.

Two weeks later, although the patient was in good general health except for difficulty in walking independently, contrast‐enhanced MRI showed enhancing effects from the brain surface along the spinal cord, with the tumor disseminated throughout the entire CSF space (Figure 5). No non‐pulmonary or new pulmonary metastases were observed. The tumors were aggressive and refractory to treatment. We introduced home healthcare and transitioned her to palliative care according to the parents' wishes. The patient was discharged home and died 3 weeks later, 15 months after the initial diagnosis.

## 3 DISCUSSION

Here, we describe a case of ATRT with lesions limited to the spinal cord at initial presentation. The patient achieved CR after chemoradiotherapy, including local irradiation and high‐dose chemotherapy, and the neurological findings temporarily improved. However, the condition exhibited progression in the CSF region, resulting in the development of cerebellar, cerebral, and spinal tumors, and the patient subsequently presented with pulmonary metastases.

Considering ATRT in spinal cord tumors is important for prompt treatment. Although spinal cord tumors are rare in children, malignant spinal cord compression is an urgent complication of pediatric spinal cancer. Prompt diagnosis and appropriate treatment are required to minimize morbidity.^12^ ATRT of the spinal region is an uncommon neoplasm that accounts for 1%–2% of all ATRTs.^13^, ^14^ Tumors can arise at any level of the spinal cord, as in intramedullary, paraspinal involvement, or any combination of these.^14^, ^15^ Its appearance is heterogeneous and may be mistaken for other more common spinal tumors, potentially delaying its diagnosis and treatment.

Researchers have attempted various treatments for ATRTs,^3^ but no standard approach has been established for pediatric ATRTs. Aggressive multimodal treatment approaches, including surgery, systemic chemotherapy with high‐dose chemotherapy, and radiotherapy, resulted in poor survival rates in ATRT patients. There are few systematic reports on the treatment of spinal ATRTs. A review of cord ATRTs in 18 patients revealed that the overall mean survival time was 10 months.^5^ A case series and literature review reported 4 cases of spinal ATRT and 47 cases of primary spinal pediatric ATRT.^7^ Three of the four patients were treated with the Dana–Faber Cancer Institute (DFCI) ATRT protocol^16^ and survived for 8.5, 22, and 45 months, respectively; one patient survived 19 years without proven INI‐1‐negative pathology, one patient experienced recurrence after completion of chemotherapy but before radiotherapy, one patient relapsed 6 months after completion of therapy, and one patient had multiple lesions in the spinal cord at initial presentation. A study of spinal ATRT in a European cohort revealed that 2 of 13 patients receiving the EU‐RHAB protocol were alive (in CR) at 69 and 72 months after diagnosis, and 1 patient was alive with distant metastatic relapse at 93 months after diagnosis.^6^ It is unclear whether local or cerebrospinal irradiation is better for radiation therapy. Considering neurodevelopment in children, local irradiation is ideal. Because our patient did not have evidence of CSF dissemination at the time of diagnosis, the patient received focal irradiation. However, given that the patient was 5 years old and that recurrence occurred in non‐irradiated fields, craniospinal irradiation at the initial onset could have prevented recurrence.

CNS tumors rarely metastasize to extra‐CNS lesions^17^; however, our patient developed extra‐CNS metastases. A summary or minireview of the WHO 2021 classification of CNS tumors made little mention of extra‐CNS metastases of ATRT.^8^, ^9^ Rhabdoid tumors that arise in intracranial locations are called ATRT, and those that arise in extracranial locations are called malignant rhabdoid tumors (MRT). ATRT comprises three main molecular subgroups: ATRT‐SHH, ATRT‐TYR, and ATRT‐MYC.^18^, ^19^ Spinal ATRT was included almost exclusively in the MYC group. Recent reports have highlighted the similarities between MRT and ATRT–MYC in terms of DNA methylation levels.^20^, ^21^ Thus, spinal ATRT may be considered part of MRT. MRT is characterized by the inactivation of the tumor suppressor gene *SMARCB1* ^22^, ^23^ and the loss of *SMARCB1* results in the upregulation of EZH2^24^, ^25^ which is responsible for the methylation of lysine 27 of histone H3 (H3K27me). EZH1 is also involved in abnormal H3K27 methylation,^26^, ^27^ and EZH1/2 dual inhibitors markedly suppressed tumor growth with no significant adverse effects in vivo.^11^ A phase I investigator‐initiated study of DS‐3201b in pediatric, adolescent, and young adult patients with malignant solid tumors (ELEPHANT trial, NCCH1904/MK007 trial) is undergoing. Our patient was unable to participate in the study because the patient did not meet the eligibility criteria. Specifically, the patient's Lansky score was less than 50. If available, the use of EZH1/2 inhibitors in addition to conventional multidisciplinary therapy may provide better treatment for patients. In addition, our patient developed extracranial metastases and treatment resistance, it may be better to consider rhabdoid predisposition syndrome, genetic and epigenetic profiling, and exploration of germline mutations in genes associated with rhabdoid predisposition syndrome (e.g., *SMARCB1* or *SMARCA4*) could provide valuable insights.^20^, ^28^, ^29^ Unfortunately, we could not perform such evaluations in the present case because the parents did not want them.

In conclusion, it is important to consider ATRT for prompt multidisciplinary treatment when presenting with a spinal tumor and the possibility of extracentral metastasis or MRT when presenting with spinal ATRT.

## AUTHOR CONTRIBUTIONS

**Akinori Yaguchi:** Conceptualization (lead); data curation (lead); formal analysis (lead); methodology (lead); writing – original draft (lead); writing – review and editing (lead). **Junya Fujimura:** Conceptualization (equal); data curation (equal); formal analysis (equal); methodology (equal); supervision (equal); writing – review and editing (equal). **Kazutaka Maruyama:** Data curation (equal); formal analysis (equal); writing – review and editing (equal). **Megumi Fujiwara:** Data curation (equal); formal analysis (equal); writing – review and editing (supporting). **Takeshi Ishibashi:** Data curation (equal); formal analysis (equal); writing – review and editing (supporting). **Osamu Tomita:** Data curation (equal); formal analysis (equal); writing – review and editing (supporting). **Toshiaki Shimizu:** Conceptualization (equal); methodology (equal); supervision (lead); writing – review and editing (equal).

## CONFLICT OF INTEREST STATEMENT

The authors declare no conflicts of interest.

## ETHICS STATEMENT

Written informed consent was obtained from the patients' guardians to publish the case details and use the images. The case discussed in this manuscript does not include patient‐identifying information, nor does it report a new study that requires IRB approval.

## Data Availability

Data sharing is not applicable to this article as no new data were created or analyzed in this study.
